# Assessment and management of pain in chronic wounds: a national survey of Australian health care practitioners caring for people with chronic wounds

**DOI:** 10.1186/1757-1146-4-S1-O16

**Published:** 2011-05-20

**Authors:** Nicoletta Frescos, Rhonda Nay, Deirdre Fetherstonhaugh, Stephen Gibson

**Affiliations:** 1 Australian Centre for Evidence Based Aged Care, La Trobe University, Bundoora Victoria, 3086 Australia; 2 National Ageing Research Institute, Parkville, Victoria, Australia

## Background

Pain is one of the key characteristics which distress patients with chronic wounds. Chronic wound pain has a significant impact on the patient’s quality of life and delays wound healing. Assessment and management of pain during wound dressing changes is well addressed, however, chronic persistent wound pain is under-assessed and under-treated resulting in patients’ perception that wound pain is something they have to suffer or manage themselves. The purpose of this study was to investigate wound practitioners assessment of chronic persistent wound pain. It sought to identify what assessment tools are utilised in determining the level of pain, the frequency of assessment and how the pain is managed.

## Methods

A national mail survey of members of the Australian Wound Management Association was conducted. The 54% response rate included nurses, podiatrists and doctors. A cross sectional descriptive analysis will be conducted of the results.

## Results

Preliminary results showed that health professionals do ask patients about wound pain mostly during wound dressing changes. Methods routinely used to assess pain were by talking to the patient or pain rating scales. Several major themes emerged linking wound pain and limitations in providing effective wound pain management.

## Conclusion

This study identified that various methods of assessment are utilized to identify pain in chronic wounds, however barriers exist that impact the implementation of effective pain management.

